# A Novel Richardson-Lucy Model with Dictionary Basis and Spatial Regularization for Isolating Isotropic Signals

**DOI:** 10.1371/journal.pone.0168864

**Published:** 2017-01-12

**Authors:** Tiantian Xu, Yuanjing Feng, Ye Wu, Qingrun Zeng, Jun Zhang, Jianzhong He, Qichuan Zhuge

**Affiliations:** 1 Institute of Information Processing and Automation, Zhejiang University of Technology, Hangzhou, Zhejiang, China; 2 Zhejiang Provincial Key Laboratory of Aging and Neurological Disorder Research, Wenzhou Medical University, Wenzhou, Zhejiang, China; University of North Carolina at Chapel Hill, UNITED STATES

## Abstract

Diffusion-weighted magnetic resonance imaging is a non-invasive imaging method that has been increasingly used in neuroscience imaging over the last decade. Partial volume effects (PVEs) exist in sampling signal for many physical and actual reasons, which lead to inaccurate fiber imaging. We overcome the influence of PVEs by separating isotropic signal from diffusion-weighted signal, which can provide more accurate estimation of fiber orientations. In this work, we use a novel response function (RF) and the correspondent fiber orientation distribution function (fODF) to construct different signal models, in which case the fODF is represented using dictionary basis function. We then put forward a new index *P*_iso_, which is a part of fODF to quantify white and gray matter. The classic Richardson-Lucy (RL) model is usually used in the field of digital image processing to solve the problem of spherical deconvolution caused by highly ill-posed least-squares algorithm. In this case, we propose an innovative model integrating RL model with spatial regularization to settle the suggested double-models, which improve noise resistance and accuracy of imaging. Experimental results of simulated and real data show that the proposal method, which we call iRL, can robustly reconstruct a more accurate fODF and the quantitative index *P*_iso_ performs better than fractional anisotropy and general fractional anisotropy.

## Introduction

Magnetic resonance imaging (MRI) can offer important insights into brain disease [1]. Only diffusion-weighted MRI (DW-MRI) can provide a unique, non-invasive technique to study the microscopic structure of brain white matter (WM) in vivo [2–4]. DW-MRI provides valuable information about the fiber architecture of tissue by measuring the diffusion of water in three-dimensional space [5, 6]. An early form of this technique, i.e., diffusion tensor imaging (DTI) [7], is widely used in clinics and provides fiber orientations of WM based on principal eigenvector of that tensor [8] and many useful quantitative indexes, including mean diffusivity (MD), fractional anisotropy (FA) [9, 10], and so on. The major shortcoming of the representative DTI is its inability to characterize the orientations of crossing and branching neural tracts in brain, especially fiber tracts with intersected diffusion orientations or partial volume averaged within a voxel [11–13]. Many recent high angular resolution diffusion imaging (HARDI) techniques have been proposed to recover the complex WM geometry [14]. Most of these methods consider water-molecule diffusion as a function of direction, such as Q-ball imaging (QBI) [15], diffusion spectrum imaging (DSI) [16] and spherical deconvolution (SD) [17], which have all conquered the limitations of DTI. However, the data acquisition times for QBI and DSI are exorbitant [18] because of the high sampling numbers required to construct the full diffusion propagator. Given the linearity and sensitivity to multi-model diffusion [11], considerable interests have been generated with the model-free SD, which is based on convolution between fiber response function (RF) and fiber orientation distribution function (fODF). Although the SD shows both good angular resolution and short computational time, the defects emerge when facing PVEs and the imaging quality is degraded by spurious directions and negative orientations caused by the truncation of high-order harmonics and ill-posed solution, even in noise-free data [19].

Partial volume effects (PVEs) were put forward by Timo Roine et al. firstly [20]. It usually appears on the border of different tissues. The brain contains complex WM and non-WM tissues, such as gray matter (GM) and cerebrospinal fluid (CSF), which have different diffusion properties. Thus, the PVEs phenomenon is particularly obvious in human brain [12, 21, 22]. For PVEs, the SD method induces some changes on RF, but this does not solve the PVEs in essence. An informed constrained spherical deconvolution (iCSD) has been proposed to improve the estimation of fODF under non-WM PVEs by modifying RF to account for non-WM PVEs locally [23]. However, the iCSD method can’t correctly resolve fiber crossing angles of less than 60° under significant non-WM PVEs. Some authors have included an isotropic compartment in their signal models but these methods both require multiple *b*-value acquisitions and distinguish the signal of different tissues [24]. In other methods based on spherical deconvolution, the isotropic signal is dampened by using an iterative RL deconvolution algorithm [25]. Falvio et al. [19] infer that fODF can be represented by several discrete Dirac delta functions on unit sphere and propose a new spherical model based deconvolution approach to solve fiber crossing. They consider isotropic diffusion and anisotropic diffusion signal and combine both of two components. Dell’Acqua et al. suggest a new term, fiber orientation function (FOF) to represent the weights of anisotropic and isotropic diffusion [26]. However, the FOF, as a combination of anisotropic and isotropic diffusions, can’t really take them apart. Consequently, The use of FOF is difficult. Isotropic signal existing in GM or CSF misleads the algorithms to produce spurious peaks in FOF. In this framework, Dell’Acqua et al. further combine RL spherical deconvolution algorithm with an adaptive regularization technique to yield damped Richardson-Lucy (dRL) algorithm in spherical deconvolution, aiming to attenuate isotropic signal while reducing spurious and non-physical fiber orientations in regions affected by PVEs [27]. dRL has its limitations. Given the different degrees of attenuation in each voxel, small FOF portions are more likely to be preserved in a low isotropic volume fraction, which leads to spurious fiber orientations [26]. Notably, the method based on RL has settled the highly ill-conditioned problem of least squares algorithm. However, in the absence of constrains of solution, even small changes in the acquired signal (e.g., MR noise) can lead to nonphysical results [17, 28]. A number of regularization algorithms have thus been developed. Yap et al. [29] develop a spatially non-negative sparse representation framework and then present an algorithm for solving *l*_0_ sparse group representation problem and apply it to tissue signal separation problem [30]. While the computational cost and intractable computation will arise when the models are more sophisticated. To make full use of spatially constraints of brain fibers, many global tractography methods considered PVEs [4, 31]have been proposed in the last two years. But there are always many disadvantages, including computing space occupied, convergence property, sub-optimal solution and so on. There is a long way to realize global tractography perfectly.

In this study, we consider a new spherical deconvolution model (hereafter denoted as iRL), which can effectively isolate isotropic signal from each DW signal. A new quantitative index is put forward to distinguish WM and non-WM of human brain, and the quantitative results of that index are better than those FA and GFA. We also propose a novel method, based on RL to efficiently reconstruct the above fiber architecture and yield high-quality fODF results. The true fODFs are gathers of delta function pointing along fiber orientations, and zero in all other orientations [32]. Thus, a dictionary basis is introduced to represent the fODF, which effectively helps to separate isotropic signal and renders the coefficients of fODF sparse. Finally, we integrate total variation regularization and *ℓ*_1_ norm regularization on the above framework to smooth noise and suppress spurious fiber orientations at the same time. To compare the performances with existing methods, the experiments are conducted on simulated and real data using the proposed method in compared with several kinds of methods, which are introduced in detail in the following sections.

## Materials and Methods

### Spherical deconvolution

Spherical deconvolution based on a relatively simple model of signal generation has been recently developed to overcome the limitations of diffusion tensor model in resolving multiple fiber orientations and to improve tractography reconstruction. The motivation of this proposed method is to recover fODF directly from DW signal without prior assumption or estimation about the number of fibers representing the information about diffusion [31]. The DW signal *S* can be assumed as a superposition of anisotropic and isotropic signal, which can be regard as two different diffusion models for three reasons. Firstly, the sampling voxels have a relatively large volume. On the border of WM and non-WM, the signal of each WM is affected by isotropic signal from non-WM, such as GM and CSF, which is known as PVEs phenomenon. The second is that isotropic diffusion exists in WM. Given that isotropic diffusion is weaker than anisotropic diffusion in WM, the diffusion in WM is always considered as anisotropy. The third one is that the complex structures of fibers such as orthogonal fibers lead to increased isotropy. Generally, the signal contributed by isotropic tissue is usually not included in spherical deconvolution models [21]. However, to facilitate calculation, researchers often try not to differentiate between the two parts and instead only make some changes in RF. The best solution is to put the two parts of DW signal segregated. In this work, we try to separate the two different parts of DW signal which would produce better imaging results especially in the DW signal existed PVEs.

Let $S^{2}$ be unit sphere domain and *SO*(3) be rotation group in $R^{3}$. The anisotropic diffusion signal is modeled by convolution between a kernel *R* ∈ *L*^2^(*SO*(3)) and a function $f\in L^{2}\left(S^{2}\right)$, which respectively represent the signal response function (RF) and fODF ideally composed of *N* Dirac delta functions for *n* bundles of fibers [33]. We assume that the isotropic signal in each voxel is the same, thus the spherical deconvolution operator can be expressed as:

$$\begin{array}{c} S\left(g\right)-\hat{S}=\int_{S^{2}}R(g\cdot v)f(v)dv \end{array}$$ (1)

where *g* are diffusion gradient orientations containing *I* directions and ${\left\{g_{i}\right\}}_{i=1}^{I}$, *S*(*g*) are diffusion attenuation signal along *g*, $\hat{S}$ are isotropic signal, which are equal along each gradient orientation and overlooked in most medical imaging cases, the dot stands for standard (Euclidean) dot product in $R^{3}$, *v* is unit sphere (*v* also represent the discretized directions of unit sphere in the following parts), *R*(*g* ⋅ *v*) is the RF describing DW signal intensity and *f*(*v*)*dv* is probability measure used to model fODF over $S^{2}$ [31]. The fODF contains all desired anisotropic information regarding both orientations of various fiber populations that may be presented and their respective volume fractions [34]. A common case is that we have *N* fibers in one voxel, where *N* is a limited natural number, and the corresponding fODF is the sum of *N* Dirac delta functions on sphere weighted by corresponding volume fractions. The form of fODF enables the separation of two diffusion models. Regarding the anisotropic signal, RF and fODF are defined as usual.

### The novel fODF estimation with double models

Basser et al. [7] indicate that the signal in a pulsed gradient spin echo depends on diffusion sensitive coefficient *b* and diffusion tensor *D*, the relation is:

$$\begin{array}{c} S\left(g_{i}\right)=e^{-tr(bg_{i}^{T}Dg_{i})} \end{array}$$ (2)

This relation relies on assumptions that the compartments have equal relaxation rates and water density, and the exchange between volumes can be neglected on the time scale of measurement [35]. Where *S*(*g*_*i*_) denotes the diffusion attenuation signal along *i*-th diffusion gradient orientation *g*_*i*_. *D* is diffusion tensor, which describes the simplest model of diffusion in axon fiber bundles. The value of *D* is the extremum direction of diffusion, which can decide the degree of water diffusion. The RF [36–38] derived from the above signal relation has a certain inaccuracy. Improving the precision of RF is of great advantage in the subsequent RL iterative model. Thus, we use the original Eq (2) as our RF.

Our final goal is to construct the fODF which characterizes the relative likelihood of water diffusion along a given direction. Most of HARDI methods do not account for PVEs caused by non-WM tissues and orthogonal fibers. Signals contributed by GM or CSF both are actually isotropic compartments and are included in the existing model of spherical deconvolution. To accurately reconstruct brain connections from DW signal, we should properly model the different types of water diffusion signal [39]. In order to make calculate easy, we discretize the process of spherical deconvolution (the discretized directions are still expressed as *v*). The reconstruction of SD method is computed as linear combination of the diffusion measurements [11]. The fODF can be reasonably considered as two main terms, *viz*. anisotropic and isotropic parts. Thus, incorporating these contributions by using double models is possible. Based on algebraic theory, we can combine the parts of anisotropy and isotropy. Thus at each voxel, the special deconvolution can be expressed as:

$$\begin{array}{c} S\left(g\right)=\hat{R}\left(g\cdot v\right)f\left(v\right) \end{array}$$ (3)

where *v* are unit direction vectors which are acquired by averaging discretization of unit spherical surface along *J* directions and ${\left\{v_{j}\right\}}_{j=1}^{J}$, $\hat{R}\left(g\cdot v\right)=\left[R_{ani}\left(g\cdot v\right)\;\;\;\;R_{iso}\right]$ and $R_{ani}{\left(g\cdot v\right)}^{\left(ij\right)}=S_{0}e^{-tr(bg_{i}^{T}D_{1}g_{i})}$, *D*_1_ is diffusion tensor of fibers (*FA* = 1, *MD* = 0.0007*mm*^2^/*s*), whose value is to ensure the maximum anisotropy, *R*_*ani*_(*g* ⋅ *v*)^(*j*)^ is the RF along *j*-th sample direction *v*_*j*_, which is a disc-shaped RF generated by the model presented in Eq (2) for a single fiber. There are *J* RFs oriented along each sampling direction. Thus, *R*_*ani*_(*g* ⋅ *v*) is an *I* × *J* matrix, *R*_*iso*_ = *S*_0_ *e*^−*tr*(*bg*^*T*^*D*_2_*g*)^ is a column vector of length *I* containing the signal of isotropic compartment. *D*_2_ is isotropic tensor of DW signal (*FA* = 0, *MD* = 0.0007*mm*^2^/*s*). Thus, the final RF $\hat{R}$ is an *I* × (*J* + 1) matrix. Naturally, fODF can be expressed as *f*(*v*) = [*f_ani_ f_iso_*], and it consists of two parts, the first *J* rows *f*_*ani*_ stand for the anisotropy. The last row *f*_*iso*_ provides information related to isotropy. The fODF can be expressed more clearly as *f* = *f*(*v*) = [*f*_*ani*_(*v*_1_), *f*_*ani*_(*v*_2_),…*f*_*ani*_(*v*_*J*_), *f*_*iso*_].

To simplify the numerical solution, the fODF constructed by SD is originally formulated using spherical harmonics basis. Actually, the proposed method can be implemented using a number of well-characterized dictionary basis sets, which are flexible unimodal basis functions. This relationship can be expressed as:

$$\begin{array}{c} f(v)=\Phi (v,u)c(v) \end{array}$$ (4)

where *u* are unit direction vectors along *L* (with *L* ≥ *J*) directions and ${\left\{u_{l}\right\}}_{l=1}^{L}$, which are used to increase the accuracy of fiber directions, Φ(*v*, *u*) is a (*J* + 1) × (*L* + 1) matrix which will be illustrated in the next step. *f*(*v*) and *c* = *c*(*v*) denote (*J* + 1) × 1 and (*L* + 1) × 1 column vectors composed of estimated values of fODF and the coefficient of fODF, respectively. Notably that the diffusion measurements *c* also consist of two parts, the first *L* rows *c*_*ani*_ show the information about anisotropy; the last raw *c*_*iso*_ represents the information related to isotropy. We can use this variable denoted as *P*_iso_ to quantify the intensity of isotropy of each voxel. *P*_iso_ can take place of the value of FA and GFA as well as conveys the message even better than them to some extent. Removing the isotropic part of each voxel inevitably increases the accuracy of fiber imaging. Once we have acquired the diffusion signal *S*(*g*) and $\hat{R}\left(g\cdot v\right)$, the unknown part fODF *f* can be computed using the iRL model.

### Dictionary basis representation

SD has been proven to produce a good imaging result. [17] proposed to express SD directly in spherical harmonics (SH) domain, so the operation can be reduced to a simple set of matrix multiplications. Simultaneously, the presence of SH basis in the process of SD has been proven to be of great importance. From a signal processing perspective, high-order SH basis is needed if we want to represent or reconstruct crossing fibers accurately with really small separated angles. However, the higher harmonic components are more sensitive to noise. Considering numerical difficulties, typically spherical harmonic up to the order of eight is used, which limits their capability in reliably resolving fiber crossing of small angles [40]. An inverse relationship exists between high frequency term and angular resolution. Thus, we cannot obtain the highest resolution and the best resistance to noise simultaneously.

**Fig 1:**
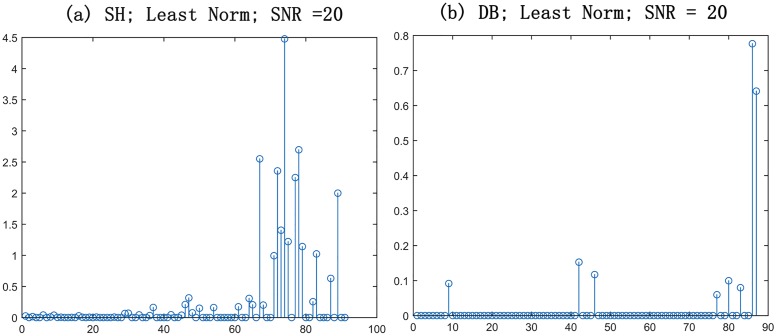
Weighting distributions. Distributions of weighting estimated by solving the Least Norm method with Spherical harmonics (SH) and Dictionary basis (DB).

On account of the above defects of SH basis, we use a new double-lobe basis function to build an over-completed dictionary basis. In this work, a set of over-completed orientation distribution basis {*d*(*v*, *u*_*l*_)|*l* = 1,…*L*} with discrete direction sets *v* ∈ *R*^*J*^ and positional direction sets *u* ∈ *R*^*L*^ are introduced to represent fiber architecture in a voxel. The basis functions are uniformly distributed in unit sphere, thereby creating a predefined fODF field. A linear weighted combination of basis can be represented as *ϕ* = [*d*(*v*, *u*_1_),…, *d*(*v*, *u*_*L*_)]. By introducing an over-completed dictionary with cardinality *L* which is larger than unit sampling direction vectors *J*, we can construct a wide-ranged basis to map the fODF. Generally, fODF can be sparsely represented by the dictionary. Hence, most of the coefficients *c* are zero. The novel basis function quoted by [41] is proposed to establish the over-completed dictionary:

$$\begin{array}{c} d\left(v,u_{l}\right)=\kappa_{1}{\left(\frac{\sin\vartheta_{v,u_{l}}}{1-\kappa_{2}\cos^{2}2\vartheta_{v,u_{l}}}\right)}^{\tau } \end{array}$$ (5)

where *ϑ*_*v*, *u*_*l*__ represent intersected angles between *v* and *u*_*l*_, and the other parameters, *κ*_1_, *κ*_2_ and *τ* are used to normalize the novel basis function. Detailed interpretation and specific parameters setting are described in [41]. Thus, we can obtain a novel dictionary basis which avoids high order’s truncation of SH function and guarantees the sparsity of coefficient at a certain extent(Fig 1). To make dictionary basis be applicable to above isotropic model, we have to make some deformations on the dictionary. The final dictionary basis can be represented as $\Phi =[\begin{array}{c} \phi \;\;\;\;\;\;\;\;\;1\\ 1^{T}\;\;\;1 \end{array}]$, where **1** represent *J* × 1 column vectors composed of 1.

### A new Richardson-Lucy model

RL model is usually used in the field of astronomical imaging. This method has two advantages: the one is that it avoids the appearance of negative values in solutions because it satisfies non-negativity constraint of solution inherently; the other is that it well controls the instabilities in the process of solving and reduces the presence of noise artifacts in the solution for its robustness to noise [19]. Thus, the RL model has already been prevalent to settle the problem of fiber imaging in neurosciences field, as originally proposed by Daube-Whitherspoon and Muehllehner in [42].

#### Richardson-Lucy model with dictionary basis

The RL model, also known as expectation maximization (EM) algorithm, follows a statistical Bayesian approach to deconvolution problem and implements an iterative estimation scheme for approximating the solutions of a maximum-likelihood problem in the case of different noises [19]. Therein, to establish a necessary foundation for the presentation and development of the proposed method, a brief overview of RL model is provided firstly. Like common approach of image restoration uses a probabilistic framework: given a sampling degraded image *S*, we can obtain the best image $\bar{S}$ (actually is the fODF) when maximizing the probability of sampling image *S*. The probability $P(\bar{S}|S)$ obeys Bayes’ rule: $P\left(\bar{S}|S\right)=P\left(S|\bar{S}\right)P\left(\bar{S}\right)/P\left(S\right)$. The magnitude signal of MR data is considered as Rician distribution [43], the likelihood is then:

$$\begin{array}{c} P\left(S|\bar{S}\right)=\frac{S}{\sigma^{2}}\exp\left\{-\frac{1}{2\sigma^{2}}\left(S^{2}+{\bar{S}}^{2}\right)\right\}I_{0}\left(\frac{S\bar{S}}{\sigma^{2}}\right) \end{array}$$ (6)

where *I*_0_ is the modified Bessel functions of the first kind of zero order, $\bar{S}$ denotes the true magnitude signal intensity in the absence of noise, *S* is measured value of each voxel with noise, and *σ*^2^ is the variance of noise. When the Rician distribution is acquired with large SNR(i.e.,$\bar{S}/\sigma \geq 3$), the process is better known as Gaussian approximation [19, 44, 45].

$$\begin{array}{c} P\left(S|\bar{S}\right)\approx \frac{1}{\sqrt{2\pi \sigma^{2}}}e^{-{\left(S-\bar{S}\right)}^{2}/2\sigma^{2}} \end{array}$$ (7)

Considering the premise of hypothesis that the sampling signal submitted to spherical deconvolution, we optimize the Eq (7). The RL model finds *f* from the observation *S*, knowing response function $\hat{R}$ by maximizing the likelihood distribution. The result can be derived by minimizing the function $-\text{log }P\left(S|\bar{S}\right)$. We suppose that noise is independent from one voxel to another. When consider the whole brain region, the log-likelihood becomes a summation of the likelihood of all voxels. The multiplicative-type algorithm is equivalent to minimize *J*_1_(*f*) given by

$$\begin{array}{c} J_{1}\left(f\right)=\sum_{x}\left(\frac{1}{2\sigma^{2}}{\left(S\left(x\right)-\hat{R}f\left(x\right)\right)}^{2}-\log\frac{1}{\sqrt{2\pi \sigma^{2}}}\right) \end{array}$$ (8)

where **x** is the voxel index. Given that the function *J*_1_(*f*) is convex in *f*, looking for a minimum is equal to searching for a zero value of gradient of *J*_1_(*f*). We set the derivative of *J*_1_(*f*) with respect to *f* to be zero and get $\sum_{x}\frac{\left(S\left(x\right)-\hat{R}f\left(x\right)\right)}{\sigma^{2}}{\hat{R}}^{T}=0$. There are some mathematical operations which can be founded in [46]. Using the dictionary basis Φ to represent the fiber orientation *f* = Φ*c*, we get:

$$\begin{array}{c} \frac{{\hat{R}}^{T}S}{{\hat{R}}^{T}\hat{R}\left(\Phi c\right)}=1 \end{array}$$ (9)

Richardson and Lucy suggested a multiplicative iterative method to solve Eq (9)

$$\begin{array}{c} c^{k+1}=c^{k}\frac{{\hat{R}}^{T}S}{{\hat{R}}^{T}\hat{R}\left(\Phi c^{k}\right)} \end{array}$$ (10)

#### Regularization with coefficient of fiber orientation

For obvious reasons, the operation of spherical deconvolution is a NP-hard problem. To render the reconstruction perfectly and stably, we use regularization on the coefficient of fiber orientation, such as, total variation (TV) and sparse regularization. Putting a priori information on the coefficient of fiber orientation seems reasonable.

One of such information is spatial consistency. Despite many advantages of RL model, the fiber detail and noise interference are contradictory during the RL iteration process. This problem is generic for all maximum likelihood techniques because we usually want to attempt to fit the data as closely as possible. Thus, a trade-off exists between quality of image and the degree of noise interference when using RL method. In the intravoxel fODF field, voxels within a small neighbourhood usually consist of similar signals. Thus, the fODF derived from voxels ought to have a correlation in spatial structure. The advantages of using TV regularization are that it reserves the similarity of coefficient and avoids noise amplification by smoothing to certain extent. Here, we introduce TV constraint on the coefficient of fODF of the entire brain image to solve the above problem by adding energy function $J_{reg}^{1}$, defined as:

$$\begin{array}{c} J_{reg}^{1}=\lambda_{TV}\sum_{x}\left|\nabla c_{i}\right| \end{array}$$ (11)

where *λ*_*TV*_ is the TV regularization parameter. Regularization is conducted in the entire field along each special gradient direction, which can be seen as *I* + 1 brain images. Although the images ${\left\{c_{i}\right\}}_{i=1}^{I}$ and *c*_*I*+1_ have different statistical properties, regularization processing in the neighbouring voxel is not prevented. The spatial dependence introduced by TV function promotes smooth solutions in homogeneous regions and prevents the solution from having oscillations. However the process of regulation will allow the solution to have sharp discontinuities [47, 48], we need to increase another constraints.

Sparse reconstruction method is broadly applied in the field of digital image processing. The sparsity constraint of the coefficient of fODF and the sparse recovery process lead us to estimate a sharp fODF from limited acquisitions. Notably, fiber orientation representation in the proposed basis is indeed sparse. The true distribution of fiber orientation can be considered sparse with the assumption that only a small number of elements of fODF are non-zero physically [49]. However, the introduction of TV regularization induces excessive smoothness between neighbouring voxels. To ensure each fiber sparse, the sparsity constraint is often added to fODF in spherical deconvolution problem. We make full use of *ℓ*_1_ norm to ensure the sparsity of coefficient in neighboring voxels. Here, we introduce the energy function of sparsity term $J_{reg}^{2}$, defined as

$$\begin{array}{c} J_{reg}^{2}=\lambda_{\ell_{1}}\sum_{x}\left|c_{i}\right| \end{array}$$ (12)

where *λ*_*ℓ*_1__ is the sparse regularization parameter. The two regularization terms based on maximum likelihood estimation can get the derivatives of *J*_*reg*_ with respect to *c*, which can be expressed as $\frac{\partial }{\partial_{c}}J_{reg}^{1}=-\lambda_{TV}div{\left(\frac{\nabla c}{\left|\nabla c\right|}\right)}_{|X}$ and $\frac{\partial }{\partial_{c}}J_{reg}^{2}=-\lambda_{l_{1}}{\left(\frac{\nabla c}{\left|\nabla c\right|}\right)}_{|x}$, respectively, where *div* and ∇ stand for divergence and differentiation, and **x** is voxel index indicating that regularization is conducted between voxels. The term |∇*c*| is replaced by its approximate value $\sqrt{{\left|\nabla c\right|}^{2}+\varepsilon }$, where *ε* is a small positive constant [47]. The total energy function is known as

$$\begin{array}{c} \begin{array}{c} J_{1}+J_{reg}^{1}+J_{reg}^{2}=\sum_{x}(\frac{1}{2\sigma^{2}}\left(S+\hat{R}\Phi c^{2}-\log\frac{1}{\sqrt{2\pi \sigma^{2}}}\right)+\\ \lambda_{TV}\sum_{x}\left|\nabla c_{i}\right|+\lambda_{\ell_{1}}\sum_{x}\left|c_{i}\right| \end{array} \end{array}$$ (13)

We minimize Eq (13) using multiplicative gradient-based algorithm (or equivalently using EM algorithm for penalized criterion of Eq (13) and obtain the final result defined as

$$\begin{array}{c} c^{\left(k+1\right)}=c^{\left(k\right)}\frac{{\hat{R}}^{T}S}{{\hat{R}}^{T}\hat{R}\left(\Phi c^{\left(k\right)}\right)}\times L_{1}^{\left(k\right)}\times TV^{\left(k\right)} \end{array}$$ (14)

where *c*^(*k*)^ is the estimated coefficient of fiber orientation, which is a ((*L*+1) × 1) dimension column vector at iteration *k* at voxel **x**, and $L_{1}^{\left(k\right)}$ and *TV*^(*k*)^ are the *ℓ*_1_ and TV regularization vector at iteration *k*. The element at different gradient positions *i* of *ℓ*_1_ regularized vector is computed as:

$$\begin{array}{c} {\left(L_{1}\right)}_{i}^{\left(k\right)}=\frac{1}{1-\lambda_{\ell_{1}}\times {\left(\frac{\nabla c_{i}^{{}^{\left(k\right)}}}{\left|\nabla c_{i}^{{}^{\left(k\right)}}\right|}\right)}_{\left|x\right.}} \end{array}$$ (15)

The element at different gradient position *i* of *TV* regularized vector is computed as:

$$\begin{array}{c} TV_{i}^{{}^{\left(k\right)}}=\frac{1}{1-\lambda_{TV}div{\left(\frac{\nabla c_{i}^{{}^{\left(k\right)}}}{\left|\nabla c_{i}^{{}^{\left(k\right)}}\right|}\right)}_{\left|x\right.}} \end{array}$$ (16)

Numerically, we notice that the regularization parameter should be neither too small nor too large. In the simulated experiments, we will discuss the selection of regularization parameters.

## Experiments and Results

### Experimental data

#### Simulated data

Datastes are generated assuming axially symmetric diffusion tensor profiles for each fiber population (*MD* = 0.7 × 10^−3^*mm*^2^/*s*) using a typical 81 directions sampling scheme [50]. To study the effect of each parameter separately in simulations, only one parameter at a time is varied. Details of these simulated datasets are provided in the following sections.

- **Simulated data1:** To guarantee the impartiality of comparative methods, We build the following simulated dataset which is the same with the data in [23], so that we can contrast iCSD method directly. Two crossing fibers are constructed, assuming the angle of crossing fiber is 70°, with varying PVEs values ranging from 0.1 to 1 (with a step of 0.1) and with different *b*-values of 1000 and 3000. The other dataset also reconstructs two crossing fibers, with varying crossing angles of fiber ranging from 40° to 90° (with a step of 10°) and with 50% isotropic signal. Complex Gaussian noise is added to obtain noisy signals with *SNR* = 20.
- **Simulated data2:** We create the synthetic data with two crossing fibers and different parameters which determine the imaging quality. Each simulated dataset is composed by 11 times 11 voxels whose fraction of isotropy is varied from 0.1 to 1 with intervals of 0.1 along x-axis, and SNR is changed from 10 to 30 with intervals of 2 along y-axis. The dataset is used to prove the validity of iRL to solve the PVEs under the condition of PVEs and noise changed. The representative angles are 40° and 90° between fiber populations in configurations, and the diffusion weighting *b* = 3000*s*/*mm*^2^.

#### IEEE international Symposium on Biomedical Imaging (ISBI) challenge phantom data

The second simulated dataset coming from the ISBI 2013 Reconstruction Challenge is acquired from an open-source software library (http://hardi.ep.ch/static/events/2013-ISBI/), which creates realistic phantoms in structural and diffusion MRI. The synthetic datasets consist of 27 simulated ground truths, including branching, kissing, and crossing structures with angles between 30° and 90°. The dataset contains 64 gradient directions with *b* = 3000*s*/*mm*^2^ at *SNR* = 10, *SNR* = 20 and *SNR* = 30. The fODF mapping is color-coded by the standard DTI colour scheme (red: left-right; green: front-back; and blue: up-down).

#### In vivo human brain data

Evaluation is performed using real human data which is published on Dipy (http://nipy.org/dipy/). The whole brain is covered with contiguous *2mm* slices with an in-plane resolution of 2 × 2*mm*^2^. For preprocessing of diffusion data, we use MRIcron and SPM8 toolbox. First, the DICOM images sets (.dcm) are split into NIfTI (.nii), gradient sequence (.bvecs), and sensitive coefficient (.bvals) datasets using MRIcron software, where the NIfTI dataset contains scanned sequence corresponding to the gradient sequence. DW images are acquired along 150 uniformly distributed directions using *b* = 2000*s*/*mm*^2^ and a single *b* = 0*s*/*mm*^2^ (the size of the whole brain is 81 × 106 × 76).

### Comparison metrics for phantom data

The performances of algorithms are quantified by comparing the obtained reconstructions with ground-truth. We adopt some of evaluation metrics widely used in the literatures [51–53].

- **Average angular error (AAE):** We compute the deviation between estimated fiber orientation and ground truth [54]:
  $$\begin{array}{c} AAE=\frac{1}{\left|\Omega \right|}\sum_{x\in \Omega }\sum_{h=1}^{n_{p}}\left|\arccos\left(\varepsilon_{x}\cdot {\tilde{\varepsilon }}_{x}\right)\right| \end{array}$$ (17)
  where *ε*_**x**_ is the “ground truth” and ${\tilde{\varepsilon }}_{x}$ is estimated fiber orientation, *Ω* is the local region used to compute angular error. we obtain one or more significant peaks of fODF (the number of peaks defined as *n*_*P*_) in each voxel **x** ∈ *Ω*, sum angular error of all peaks and finally get the average angular error. These operations are repeated about 100 times.
- **Average probability of false direction (APFD):** APFD is used to evaluate the probability of false directions compared with real fiber number ${\tilde{M}}_{x}$ inside a voxel **x**. The ratio of false positive (*r*^+^) and ratio of false negative (*r*^−^) are defined as
  $$\begin{array}{c} r^{+}=\frac{1}{\left|\Omega \right|}\sum_{x\in \Omega }\left|M_{x}^{+}-{\tilde{M}}_{x}\right|\cdot 100\%,\;\;r^{-}=\frac{1}{\left|\Omega \right|}\sum_{x\in \Omega }\left|{\tilde{M}}_{x}-M_{x}^{+}\right|\cdot 100\%,\;\; \end{array}$$ (18)
  In a region Ω, $M_{x}^{+}$ and $M_{x}^{-}$ denote the over-estimated and under-estimated number of fibers inside a voxel compared to ground truth.
- **Fractional anisotropy (FA):** The *FA* characterizes the degree of “out-of-roundness” of diffusion ellipsoid. It measures the fraction of total magnitude of diffusion tensor that is anisotropic
  $$\begin{array}{c} FA=\sqrt{\frac{{\left(\lambda_{1}-\bar{\lambda }\right)}^{2}+{\left(\lambda_{2}-\bar{\lambda }\right)}^{2}+{\left(\lambda_{3}-\bar{\lambda }\right)}^{2}}{2\left(\lambda_{1}^{2}+\lambda_{2}^{2}+\lambda_{3}^{2}\right)}} \end{array}$$ (19)
  where *λ*_1_, *λ*_2_, *λ*_3_ are the eigenvalues provided by diffusion tensor, which is one of the most important rotationally invariant quantitative scalar parameters. $\bar{\lambda }$ is the arithmetic mean of the three eigenvalues.
- **Generalized fractional anisotropy (GFA):** Scalar measures on the fODF are useful in defining tissue contrast, performing statistical analyses, or summarizing the geometric properties of fODF. We define the scalar measures GFA as
  $$\begin{array}{c} GFA=\frac{std\left(f\right)}{rms\left(f\right)}=\sqrt{\frac{n\sum_{j=1}^{n}{\left(f\left(v_{j}\right)-\left\langle f\right\rangle \right)}^{2}}{\left(n-1\right)\sum_{j=1}^{n}f{\left(v_{j}\right)}^{2}}} \end{array}$$ (20)
  where *n* is the number of fODF, *std* is the standard deviation, *rms* is the root-mean-square, and $\langle f\rangle =\frac{1}{n}\sum_{j=1}^{n}f(v_{j})$ is the mean of the ODF. The GFA metric is automatically normalized to [0, 1].
- **Generalized relative anisotropy (GRA):** The GRA scalar represents a measurement of deviation from the isotropic state of the fODF of each voxel:
  $$\begin{array}{c} GRA=\sqrt{\frac{\sum_{j=1}^{n}{\left(f\left(v_{j}\right)-\left\langle f\right\rangle \right)}^{2}}{n\left\langle f\right\rangle }} \end{array}$$ (21)

It’s worth noting that peaks in clusters that are less than half of the crossing angle (with an upper limit of 35 degrees) from the true orientations are considered correct peaks.

### Implementation details

All experiments of the proposed method are conducted on Inter(R)@2.4 GHz (48 G RAM, 64 bit). For measured signal, the obtained mask image is down-sampled to the dimensions of dMRI. Mask analysis is conducted on DSI Studio 1 (http://www.dsi-studio.labsolver.org). For the dictionary basis, the dimension of coefficients and the basis vectors are the same, representing the related percentage of each dictionary basis. For the positional direction sets *u* of dictionary basis, a tessellation scheme is distributed evenly on 321 points on a hemisphere and is generated by the subdivision of the face of an icosahedron. By avoiding repeated sampling, the discrete direction sets *v* are made to be identical with *u*. To perfectly reconstruct the fODF, the reconstructed dictionary basis is designed using a symmetric sphere with 10 242 vertices from Dipy (http://dipy.org/), which is an array of 10 242 fODF values corresponding to the vertices of sphere. To ensure the applicability of in vivo data, the two RFs in vivo data are acquired according to typical value of diffusion tensor signals in the corpus callosum and cortex respectively [17, 23]. We choose 50 voxels with the highest FA and use the average of signals whose principal eigenvectors are aligned along z-axis to acquire the anisotropic RF. Identically, we choose 50 voxels with the lowest FA and use the average of signals to acquire the isotropic RF.

We compare the proposed method iRL with the other state-of-the-art methods on simulated phantom and real data. The alternative approaches include Recursive calibration constrained spherical deconvolution (RC-CSD) [55], Sparse Fascicle Model (SFM) [56], damped Richardson Lucy (dRL) [26], information constrained spherical deconvolution (iCSD) [23] and Multi-shell multi-tissue constrained spherical deconvolution(MSMT-CSD) [24]. RC-CSD is an improvement of SD, which provides an accurately calibrated RF. SFM treats each MRI voxel as two types of compartments, non-oriented tissues and oriented fascicles considering the PVEs, which is implemented using Dipy (http://nipy.org/dipy/index.html) publish library [57]. The dRL is aiming at reducing isotropic background effects in spherical deconvolution, which is implemented using a software package provided in (http://neuroimagen.es/webs/hardi-tools/). The iCSD improves the estimation of fODF by modifying the RF to account for non-WM PVEs locally. MSMT-CSD uses CSD approach to estimate a multi-tissue ODF and implements in MRtrix (http://www.mrtrix.org/) [58]. It’s worth mentioning that MSMT-CSD can reconstruct brain fibers using single shell data, but the function of separating different tissues can not work well. The number of iterations of each method is set to 200 times. The related parameters used in compared methods are set to their optimal values according to the reference documents. For dRL algorithm, *η* acts as a threshold parameter and controls the damped amplitude of FOF, which is set to *η* = 0.08.

### Results

#### Optimal regularization parameter

**Fig 2:**
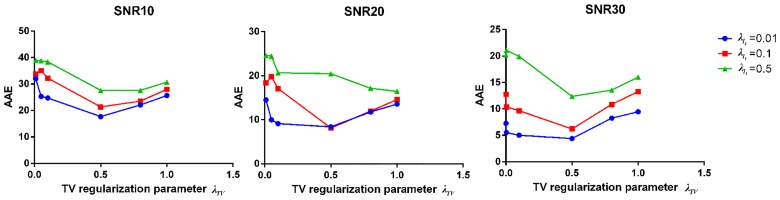
Simulated results of parameter selection. Average angular error using different regularization parameters in ISBI data with different SNRs.

The new deconvolution algorithm with TV and *ℓ*_1_ regularization has shown good imaging result with the elaborately chosen regularization parameters. The choice of good parameters value plays a crucial role in imaging results when using iRL. Thus, the first step of our experiment is to study if and how *c* estimation is influenced by setting different regularization parameters and by choosing different numbers of algorithm iterations during the process of our algorithm. To obtain the best regularization parameters and the number of iterations, we use different parameters to image the ISBI data with SNRs of 10, 20 and 30, and identify the quantitative index to evaluate image quality. To select regularization parameters, we use the AAE to be the quantitative index (Fig 2).

We performe 100 repetitions with simulated data. We find that the *ℓ*_1_ regularization parameter affects the angular resolution of imaging fiber and the TV regularization plays a vital role in resisting noise. We need only to increase the value of the TV or *ℓ*_1_ regularization parameters to improve the quality of imaging when the signals have low SNR or small angle, respectively. From Fig 2, the best regularization parameters can be set to *λ*_*ℓ*_1__ = 0.01 and *λ*_*TV*_ = 0.5. The RL algorithm has certain superiority in resisting noise, but when the SNR is low, as shown in Fig 2, the imaging results are unsatisfactory and have a relatively large angular error.

The RL algorithm is known to have the property of ‘semi-convergence’ [59], i.e., the solution initially converges to the true value and then diverges as iterations proceed [19]. We choose 200 as the maximum iteration numbers to prevent noise amplification and generation of artifacts.

#### Simulated data in the presence of isotropic diffusion

**Fig 3:**
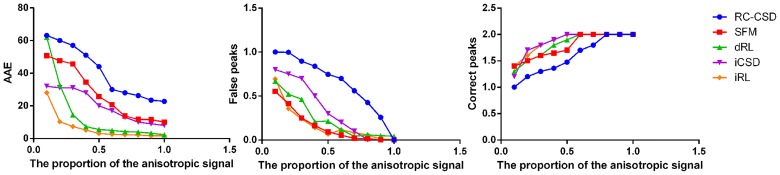
Comparison of simulated results. AAE, False peaks and Correct peaks for different proportions of anisotropic signal (diffusion weighting 3000*s*/*mm*^2^, angle 70°, and SNR 20).

**Fig 4:**
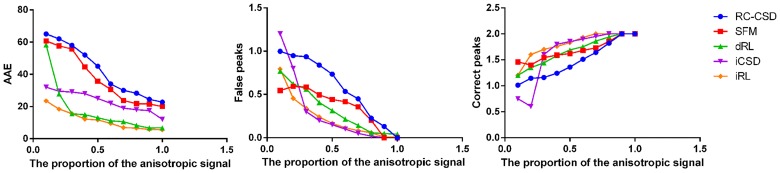
Comparison of simulated results. AAE, False peaks and Correct peaks for different proportions of anisotropic signal (diffusion weighting 1000*s*/*mm*^2^, angle 70°, and SNR 20).

We use different simulated datasets to verify the effectiveness of iRL. Comparative tests are conducted by four kinds of methods. This experiment is used to verify the ability of imaging the signal with different volume fractions of isotropic signal (Fig 3). The other simulated datasets are generated in the same way, excepting that the diffusion weighting *b* is changed (Fig 4). We perform 100 repetitions with the simulated datasets that are generated randomly.

Compared with the low *b* value dataset, the high *b* value dataset shows fODF with a partial increase in angular resolution. However, no change exists in angular resolution for the signal with low proportion of anisotropy. Figs 3 and 4 show that when the signal has high proportion of anisotropy, the imagings of five kinds of methods are all accurate. The iCSD and iRL have a relative better angular resolution and less numbers of false peaks. When high isotropy exists in the simulated signal, iRL is advantageous over the other four kinds of methods in the aspect of angular resolution. Regardless of signal composition, iRL has the best and smallest angular resolution.

**Fig 5:**
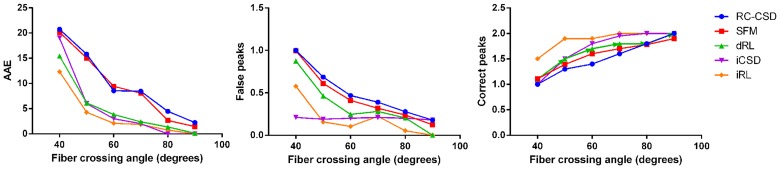
Comparison of simulated results. AAE, false peaks and correct peaks for different crossing angles(with 50% isotropic signal, diffusion weighting 3000*s*/*mm*^2^, and SNR 20).

We perform simulated experiments to investigate the simulated datasets with different fiber crossing angles. We utilize five methods to image the above simulated signals respectively. This experiment is used to verify the ability of imaging the signal with different crossing angles (the results are shown in Fig 5). We also perform 100 repetitions with the simulated datasets which are generated randomly.

The five methods are all becoming more effective as the crossing angles increasing. In our method, the quantitative indexes of AAE and false peaks is lower for all angles and the precision is improved remarkably for angles larger than 50° (Fig 5). It’s worth mentioning that the 40° crossing angle could be identified with 50% PVEs using iRL.

**Fig 6:**
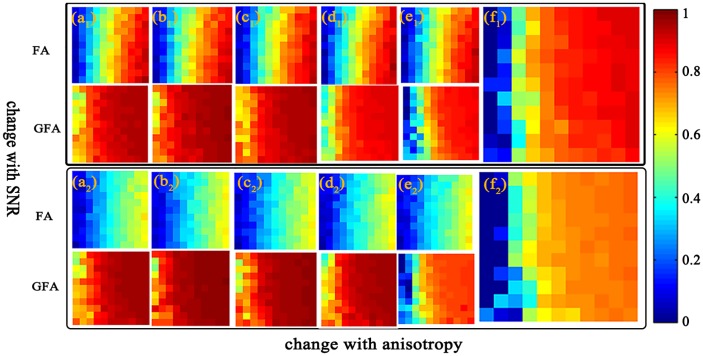
Comparison of the simulated results. FA and GFA with RC-CSD(a), SFM (b), dRL (c), iCSD (d) and iRL (e). Figures a_1_-f_1_ are the imagings for 40° cross-angle. Figures a2-f2 are the imagings for 90° cross-angle. Figures f_1_-f_2_ are the new index *P*_iso_.

We also perform simulated experiments to investigate simulated datasets with different PVEs and SNRs and utilize five methods to image the above simulated signals respectively (the results are shown in Fig 6). To verify the effectiveness of our method in aspect of the new isotropic quantitative index, we conduct the signal of simulated data2 and the imaging result is mapped to quantitative indexes, FA and GFA included.

In the case of anisotropy and SNR increased, the upper-left corner of each figure has the poorest simulated signal, and the lower-right corner of each figure has the best simulated signal. In Fig 6, we compare FA, GFA, and our new quantitative index *P*_iso_ (because the quantification of *P*_iso_ is the extent of isotropy, which is contrary to FA and GFA. For comparison, we make *P*_iso_ a reverse imaging) using different methods (Figures a_1_-e_1_) and different crossing angles (Figures a_1_-a_2_). For 40° cross fibers, no significant difference is observed. For 90° cross fibers, the quantitative index FA has an obvious deficiency in which the degree of anisotropy is lower than the normal levels. However, the quantitative indexes GFA and *P*_iso_ have a correct indication. Considering both experiments, *P*_iso_ has better implementation in low anisotropy.

#### fODF estimation for ISBI data

**Fig 7:**
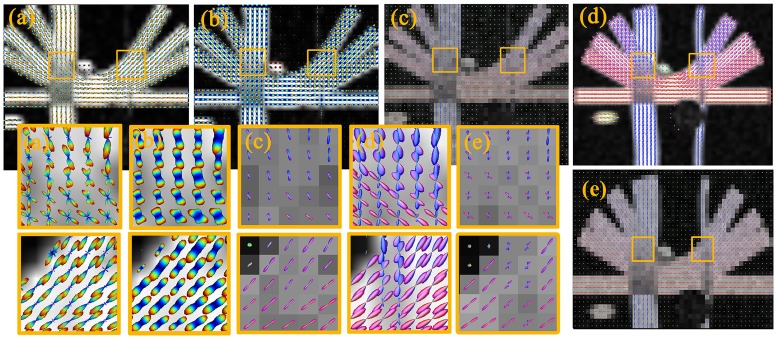
Visualization of the fODF reconstructed from ISBI dataset with HARDI data. Depicted fODF profiles correspond to estimations from the RC-CSD (a), SFM (b), dRL (c), MSMT-CSD (d) and our method iRL (e).

We compare several different methods using the authoritative ISBI simulated experiment data. Fig 7 compares the reconstructed fODF. We observe that the fODF estimations of each voxel are relatively independent and prone to noise. The fiber orientations reconstructed by standard RC-CSD, SFM, dRL, and MSMT-CSD methods always lack important information on fiber crossing.

**Fig 8:**
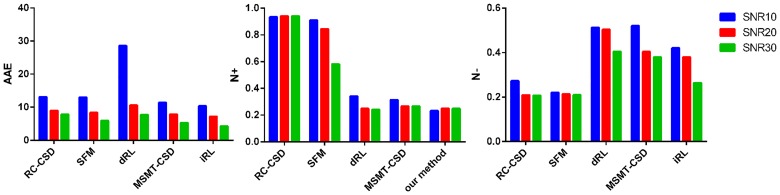
Quantification of the reconstruction accuracy. The results of RC-CSD, SFM, dRL, MSMT-CSD, and iRL in terms of *AAE*, *n*^+^, and *n*^−^ using ISBI data.

In the marked regions in Fig 7, the crossing angles are very small. The iRL can separate this part of crossing, but the results are imperfect. In the crossing fiber case, performances are assessed according to two criteria: (1) the effect of miscalibration on angular resolution, and (2) the over-estimated and under-estimated number of fibers. Fig 8 shows that iRL produces fewer angular errors. About the overestimation of false peaks, iRL has a better result when compared with RC-CSD and SFM. There is a better result about underestimation of false peaks when compared with dRL and MSMT-CSD. It’s mentioning that the iRL has fewer total numbers of false peaks than the other five methods, regardless of SNR.

#### fODF estimation for human data

Evaluation is performed using real human data acquired on public datasets (http://nipy.org/dipy/). We select two representative areas, one of the areas contains multiple functional areas of the brain, such as the cortex and CSF (i.e, containing possible isotropic compartment).

**Fig 9:**
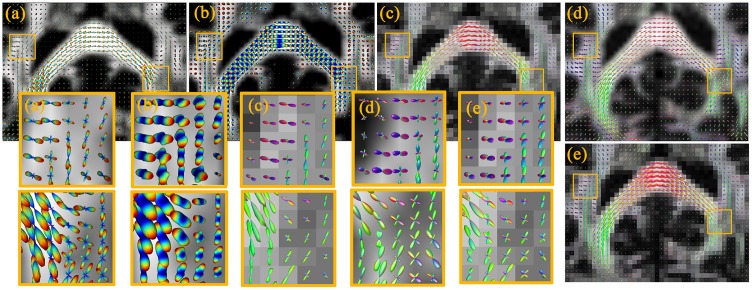
Visualization of fODFs reconstructed from real data. Depicted fODF profiles correspond to the estimations from RC-CSD (a), SFM (b), dRL (c), MSMT-CSD (d) and iRL (e). The background images are fractional anisotropy images computed from each reconstruction.

**Fig 10:**
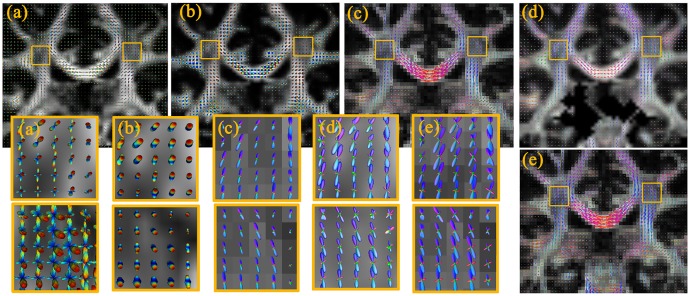
Visualization of fODFs reconstructed from real data. Depicted fODF profiles correspond to the estimations from RC-CSD (a), SFM (b), dRL (c), MSMT-CSD (d) and iRL (e). The background images are the fractional anisotropy images computed from each reconstruction.

Fig 9 compares the intravoxel fiber architecture estimated by five different methods on the human datasets. In the posterior thalamic radiation (refer to Human Brain in ICBM-152 Space) region (marked with a yellow box in Fig 9), the situation of fiber crossing is complex, containing single fiber and multiple fiber crossings. The iRL has a good imaging of multiple fiber crossing trends. The other methods always lack of some fiber directions. The same results can be seen in Fig 10. In addition, in the posterior thalamic radiation region, the isotropic signal is stronger, and the compared results are more obvious. In particular, the fibers (red ellipses) in the superior temporal gyrus WM (STG-WM) and the middle temporal gyrus WM (MTG-WM) regions are well represented by iRL.

The quantitative indexes of GM and WM are carried out in above areas. We use *P*_iso_ to quantify the difference between WM and GM in brain regions by using different indexes, including FA, GFA, and GRA. The three indexes are well-known and used in various occasions to describe the strength of anisotropic diffusion.

**Fig 11:**
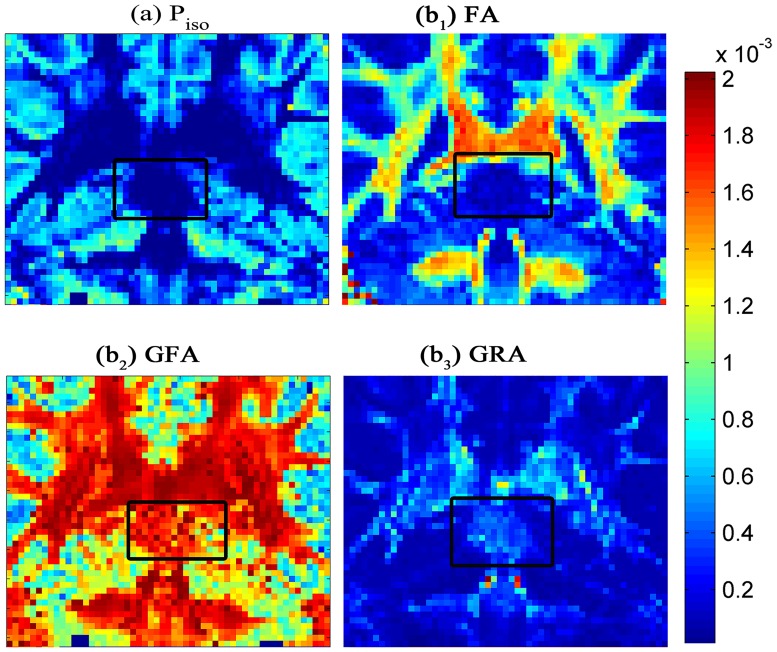
Display of diffusion degree using four methods. (a): *P*_iso_ quantifies the intensity of isotropic signal, and (b_1_-b_3_) quantify the intensity of anisotropic signal.

The degree of diffusion anisotropy is severely underestimated using the indexes calculated by diffusion coefficients acquired in fiber orientations. Some researchers present that water diffusivity in the directions parallel to the fiber is almost 10 times higher than the average diffusivity in directions perpendicular to them [9]. The marked area where the fibers have vertical distribution. The anisotropy is actually very strong, whereas the figure of FA (Fig 11b_1_) shows a strong isotropy. The figures of *P*_iso_ (Fig 11a), GFA (Fig 11b_2_), and GRA (Fig 11b_3_) show similar results on anisotropy.

## Conclusions and Discussion

We focus on PVEs in the reconstruction of fiber configuration, which rarely elicit interest of researchers. PVEs are some of the greatest obstacles in improving the accuracy of fiber imaging. We usually utilize the anisotropic signal to reconstruct fiber orientation, which is affected by the isotropic signal. Only by removing the isotropic signal from DW signal, can we obtain the best imaging results, as we have done in this paper. The contribution of our approach is that we initially propose a method based on the local maximum likelihood estimation to isolate the isotropic from DW signal in entire regions included in both non-WM and WM by rebuilding RF and fODF used to estimate the coefficients of fODF to account for tissues composition. At the same time, the separated parts can be used to quantify the degree of isotropic signal in each individual voxel. Secondly, the application of dictionary basis and RL model successfully solves the ill-posed problem and ringing effect. Finally, the spatial regularization of FOD is approximated by combining TV and *ℓ*_1_ norms that stabilize the deconvolution problem and promote sparsity in the solution. We also compare the performances of proposed method with several state-of-the-art algorithms on synthetic data and human brain datasets. Results show significant improvement over contrastive methods in its ability to reduce false positive fiber orientations and preserve angular resolution on both simulated and in vivo datasets.

Some of non-WM PVEs are due to the reduced SNR of WM compartment, which cannot be recovered, and the rest of effects are due to mostly isotropic diffusion from non-WM tissue [23]. In this paper, we extend PVEs’ influence, including the isotropic diffusion in WM and the increase in isotropy caused by complex fiber directions. By isolating the isotropic signal, the imaging results significantly improve, especially on the AAE, throughout the whole brain. From the Fig 8, we control the AAE within 8° using open ISBI data.

Simulated results show that with the reduction of isotropic signal, the AAE significantly increases. As regard 50° of crossing fiber, although the proportion of isotropic signal is as low as 0.1, the AAE remains within 30°. This is a complicated process because the imaging result is affected by many parameters, such as *b* value, regularization parameters, iterations, and so on. For different datasets, we should adjust the corresponding parameters to obtain the best imaging results. Notably, a lower *b* value leads to poorer imaging. We can find another defect, i.e., the decrease in fiber quantity is more outstanding than the overestimation of fiber in the simulated data. This problem is inherent in the method related to RL, which will be our concern in a future study.

Real experimental results indicate that iRL efficiently improves the ability of resolving crossing fibers in regions with high PVEs, whereas in high anisotropy regions, iRL and others produce roughly identical results. In the region of the internal capsule and the corpus callosum, the tracts have relatively larger amplitude, which is particularly useful in connectomics. Given the abandonment of least squares and spherical harmonic function, the spurious fODF peaks (consistent with well-known ringing artefacts) have a prominent reduction on Figs 9 and 10. The comparisons of the tract density image between iRL and others show increased tract density in the main WM tracts and decreased tract density in non-WM region, which are useful for fiber tracking.

Some open areas of researches exist in iRL. Firstly, for the two different diffusion models, different choices exist for regularization parameters. Considering the different diffusion regions, the strength of regularization should be discrepant. Secondly, a calibrated RF must be used to further reduce spurious peaks. Fortunately, the methods based on RL have a low over-all sensitivity to miscalibration. Thirdly, this method has potential to considerably reduce gradient directions, indicating a clinically feasible acquisition time. Thus, the application of this method is significant in clinical studies in the future. Finally, the assumed unimodal Gaussian diffusion model does not apply to MRI measurements, which are completely proven to be Rician distribution model [60]. These existing problems will be studied in our future work.
